# Achieving Complete Radiological and Bio-Chemical Response as a Predictor of Long-Term Survival in Stage IV Epithelial Ovarian Cancer

**DOI:** 10.7759/cureus.20017

**Published:** 2021-11-29

**Authors:** Hafiz Abubakar Sarwar, Jhanzeb Iftikhar, Musa Azhar, Kiran Munawar, Muhammad Rashid Hanif, Muhammad Abu Bakar, Neelam Siddiqui

**Affiliations:** 1 Medical Oncology, Shaukat Khanum Memorial Cancer Hospital and Research Center, Lahore, PAK; 2 Medical Oncology, Shaukat Khanum Memorial Cancer Hospital and Research Centre, Lahore, PAK; 3 Biostatistics and Epidemiology, Shaukat Khanum Memorial Cancer Hospital and Research Centre, Lahore, PAK

**Keywords:** interval debulking surgery, hereditary breast and ovarian cancer, ovarian cancer, metastatic ovarian cancer, epithelial ovarian cancer

## Abstract

Objective

Epithelial ovarian cancer (EOC) is common among ovarian cancers. The majority of existing literature shows combined data of stage III and stage IV. Therefore, we aimed to look for whether achieving complete radiological and biochemical response after initial treatment of stage IV epithelial ovarian cancer as a predictor of long-term survival in the Pakistani population.

Methods

A cross-sectional study was conducted of patients with stage IV epithelial ovarian cancer diagnosed and treated from 2006-2013 at Shaukat Khanum Memorial Cancer Hospital and Research Centre, Lahore, Pakistan. Overall survival was defined as the number of months between patients’ diagnosis at the hospital and any cause of death or last follow-up date. Kaplan Meier curve was used to report the overall survival. The log-rank test was used to distinguish the survival difference in complete and no complete response. P-value <0.05 was considered statistically significant.

Result

A total of fifty patients of stage IV epithelial ovarian carcinoma, with a mean age of 53 ± 2 received neoadjuvant chemotherapy and suitable patients underwent interval-debulking surgery. Among these fifty patients, twenty-one (42%) patients who achieved complete radiological and biochemical response had a median survival of greater than five years. Patients without co-morbidities (46%) and having good performance status (52%) showed better results of the treatment. Patients’ tolerance to chemotherapy with good response and fit enough to undergo interval-debulking surgery, achieving complete radiological and biochemical response after initial induction therapy were significantly associated with long-term survival (P<0.05).

Conclusion

Outcomes of patients who present with stage IV EOC remains dismal. Patients who achieved complete radiological and biochemical response after neoadjuvant chemotherapy and interval-debulking surgery was significantly associated with long-term survival.

## Introduction

Ovarian cancer is the fifth leading cause of cancer-related death and accounts for 3% of all cancers among women worldwide [1]. Ninety percent of malignant tumours of the ovary are Epithelial Ovarian Cancers (EOC). EOC is further classified into Type I and Type II tumours [2,3] Low-grade tumours are type I tumours and carry mutations of BRAF, KRAS, and PTEN. These tumour types include endometrioid, mucinous, and clear cell histologies. While type II tumours include high-grade serous, high-grade endometrioid, malignant mixed mesodermal tumors, and undifferentiated carcinomas. These tumour types harbour BRCA1, BRCA2 and p53 mutations [4].

The majority of the patients are postmenopausal women and seventeen percent (17%) of the patients present with stage IV disease at diagnosis. A significant proportion of patients has advanced age and poor performance status and five-year survival rate remain less than 10%, and 10 years overall survival rate of only 5% [5-7]. Studies conducted by Winter and colleagues found that patients with increasing age, poor performance status, mucinous or clear cell histology, and gross residual disease after primary surgery are independent predictors of progression-free survival (PFS) and overall survival (OS) in advance stage disease [8-9]. At present, with marked improvement in treatment options and improved surgical techniques, a substantial number of patients achieve complete biochemical and radiological remission. However, patients with advanced-stage disease, relapse early after treatment completion. Platinum resistance is a major factor in patients who relapse early, while patients whose disease remains platinum-sensitive, show good clinical outcomes [3].

According to global cancer statistics, ovarian cancer will develop in 295,000 women every year worldwide and ninety percent are epithelial ovarian cancers [1]. Many studies conducted in Pakistan highlighted that its incidence is rising by one-two percent every year and it stands among the ten most common cancers in this region [10]. A study conducted in Pakistan showed that surface epithelial tumours constitute 63.5% of all tumours of the ovary. Serous cystadenocarcinoma was the commonest among malignant tumours (33.3%). Among benign tumours, benign cystic teratoma was the most prevalent (35.2% of all benign tumours) [11]. Another institutional cancer data in Pakistan revealed that breast cancer was the commonest cancer accounting for 38.5% of female malignancies followed by ovarian cancer 13.6% [12]. A study conducted at our hospital found that the majority of the patients with ovarian cancer present with advanced-stage disease [13]. Outcomes are poor in these patients due to late presentation and platinum-resistant disease and unavailability of newer targeted agents. In developed countries, like the United States, it is the fifth leading cause of cancer-related death. The majority of the women are diagnosed with advanced-stage disease and the prognosis is dismal in these patients. There is a statistically significant improvement in the five-year survival of patients with the advancement in the surgical technique and availability of newer agents.

In Pakistan, there is a lack of data regarding prognostic factors, which are associated with long-term survival particularly in stage IV disease. Secondly, the majority of existing literature shows combined data of stage III and stage IV. We aim to predict whether achieving a complete radiological and biochemical response in stage IV EOC after initial treatment is associated with long-term survival in patients who were diagnosed and treated at the Shaukat Khanum Memorial Cancer Hospital and Research Centre (SKMCH&RC), Lahore, Pakistan. 

## Materials and methods

This retrospective cross-sectional study was done at Shaukat Khanum Cancer Hospital & Research Centre (SKMCH&RC), Lahore, Pakistan. The data of all patients with histologically confirmed ovarian cancer who presented to SKMCH&RC from January 2006 till December 2013 was reviewed after acquiring approval from the institutional review board. The data was extracted electronically from the hospital information management system. A total of ninety-eight patients with ovarian cancer were identified as per TNM (Tumor, Nodes, Metastasis classification of malignant tumors)and FIGO (International Federation of Gynaecology and Obstetrics) Staging System for ovarian, fallopian tube, and primary peritoneal cancer, American Joint Committee on Cancer (AJCC) Staging System 8th edition, 2017.

The study included all the patients with stage IV epithelial ovarian cancer diagnosed and treated at our institute. Stage IV was defined according to FIGO staging and included the presence of any distant metastasis, including inguinal lymph node metastasis, liver parenchymal deposits and pleural effusion. Patients with pleural effusion had cytology by fine needle aspiration to confirm the presence of malignant cells. The patients with early-stage epithelial ovarian cancer, ovarian germ cell tumours and sex cord tumours were excluded from this study. A total of fifty patients met the inclusion criteria. The data regarding age, body mass index, comorbidities, performance status, CA-125, histopathology, site of metastasis, ascites, number of chemotherapy cycles, surgery performed, clinical response and survival was obtained. We included diabetes mellitus, hypertension, ischemic heart disease, chronic kidney disease, hypothyroidism, cerebrovascular accident, and psoriatic arthritis in comorbidities.

The end-point was time to death or last follow up greater than 36 months; patients were labelled as long-term survivors in this instance. Overall survival (OS) was defined as the time from date of registration to time of death of any cause or last follow-up.

Statistical analysis

We performed a statistical analysis of the data using SPSS software (version 23.0; IBM, Armonk, USA). Mean ± standard deviation was used for continuous variables, while frequencies and percentages were used for categorical variables. Chi-square test or Fisher's exact test (when necessary) were used to compare categorical variables. The Kaplan-Meier method was used to estimate survival as a function of time, and the log-rank test analysed survival differences. Statistical significance was defined as a two-tailed p-value 0.05.

## Results

Table 1 presents the patient’s demographic and clinical features of fifty patients of stage IV EOC with a mean age and standard deviation of 53 ± 2 years. Patients had a mean body mass index with a standard deviation of 27 ± 1. In this study population, less than half of the patients (46%) had no comorbidities, while fifty-four percent (54%) patients had one or more co-morbidities. Only eight percent (8%) of patients had an ECOG (Eastern Cooperative Oncology Group) performance status of 0 (fully active, able to carry out all pre-disease activities without restriction). Fifty-two percent (52%) of the patients had an ECOG performance status of 1 (able to carry out light housework or office work). While forty percent (40%) had an ECOG performance status of 2 or more than 2 (meaning that patients were ambulatory and capable of all self-care but unable to carry out any work activities; up and about more than 50% of waking hours), and ECOG performance status of 3 (capable of only limited self-care, confined to a bed or chair more than 50% of waking hours).

**Table 1:** Descriptive statistics (demographics and clinical characteristics) * standard error; ECOG: Eastern Cooperative Oncology Group

Variables	Categories	Total = N (%)	Alive 36 (72%)	Death 14 (28%)	p-value
Age (years)	Mean ± SE*	53 ± 2	54 ±2	49 ±4	0.25
Body Mass Index	Mean ± SE*	27 ± 1	27 ± 1	28 ± 2	0.80
Comorbid	No	23(46%)	17(47%)	6(43%)	0.78
	Single	13(26%)	10(28%)	3(21%)	
	Multiple	14(28%)	9(25%)	5(36%)	
ECOG	0	4(8%)	1 (3%)	3 (21%)	0.30
	1	26(52%)	18 (50%)	8 (57%)	
	Above 1	20(40%)	17 (47%)	3 (22%)	

Furthermore, Table 2 presents the pathological characteristics of the patients. The majority of the patients (54%) had high-grade serous histopathology followed by mucinous and adenocarcinoma. CA-125 value at diagnosis was found with a standard deviation of 3260 ± 752. In twenty-one patients (42%), the disease metastasized to pleura (pleural effusion was present M1), while in twenty-nine patients (58%), metastatic sites were liver, extra-abdominal lymph nodes, large intestine and spleen (M2). There was no statistically significant difference in patient outcomes who had either single or multiple organ metastasis. Thirty-nine (78%) patients had ascites at the presentation time, while eleven patients (22%) had no ascites. Twenty-nine patients (58%) underwent a surgical procedure (total abdominal hysterectomy + bilateral salpingo-oophorectomy + omentectomy + tumour debulking).

**Table 2:** Descriptive statistics (Pathological characteristics) * standard error; NOS: not otherwise specified *Complete Response (CR):* Disappearance of all target lesions and reduction in the short axis measurement of all pathologic lymph nodes to ≤10 mm *Partial Response (PR):* ≥30% decrease in the sum of the longest diameter of the target lesions compared with baseline *Progressive Disease (PD):* ≥20% increase of at least 5 mm in the sum of the longest diameter of the target lesions compared with the smallest sum of the longest diameter recorded *Stable Disease (SD):* Neither PR nor PD

Variables	Categories	N(%)	Alive 36 (72%)	Death 14 (28%)	p- value
CA-125 (normal value < 46 U/mL)	Mean ± SE*	3260 ± 752	3875 ± 1015	1679 ± 1767	0.19
Histopathology	Adenocarcinoma NOS	6 (12.0%)	4 (11.1%)	2 (14.3 %)	0.34
	Serous	27 (54.0%)	21 (58.3 %)	6 (42.9 %)	
	Mutinous	3 (6.0%)	3 (8.3 %)	0 (0 %)	
	Endometroid	5 (10.0%)	3 (8.3 %)	2 (14.3 %)	
	Clear cell	1(2.0%)	0 (0 %)	1(7.1 %)	
	Others	8 (16.0%)	5 (13.9 %)	3 (21.4 %)	
Site of Metastasis	M-1	21 (42%)	15 (42%)	6 (43%)	0.94
	M-2	29 (58%)	21 (58%)	8 (57%)	
Ascites	No	11 (22%)	8 (22%)	3(21%)	0.64
	Yes	39 (78%)	28 (78%)	11(79%)	
Number of chemotherapy cycles	Mean ±SE*	19 ± 3	19 ± 3	17 ± 3	0.64
Surgical Resection	Surgery Performed	29 (58%)	21 (58%)	8 (57%)	0.94
	No surgery performed	21 (42%)	15 (42%)	6 (43%)	
Response	Complete Response	21 (42%)	17 (47%)	4 (29%)	0.14
	Partial Response	20 (40%)	14 (39%)	6 (43%)	
	Stable Disease	2 (4%)	0 (0%)	2 (14%)	
	Progressive Disease	7 (14%)	5 (14%)	2 (14%)	
Survival Time (months)	Mean ± SE*	40 ± 5	41 ± 6	38 ± 8	0.74

**Figure 1:**
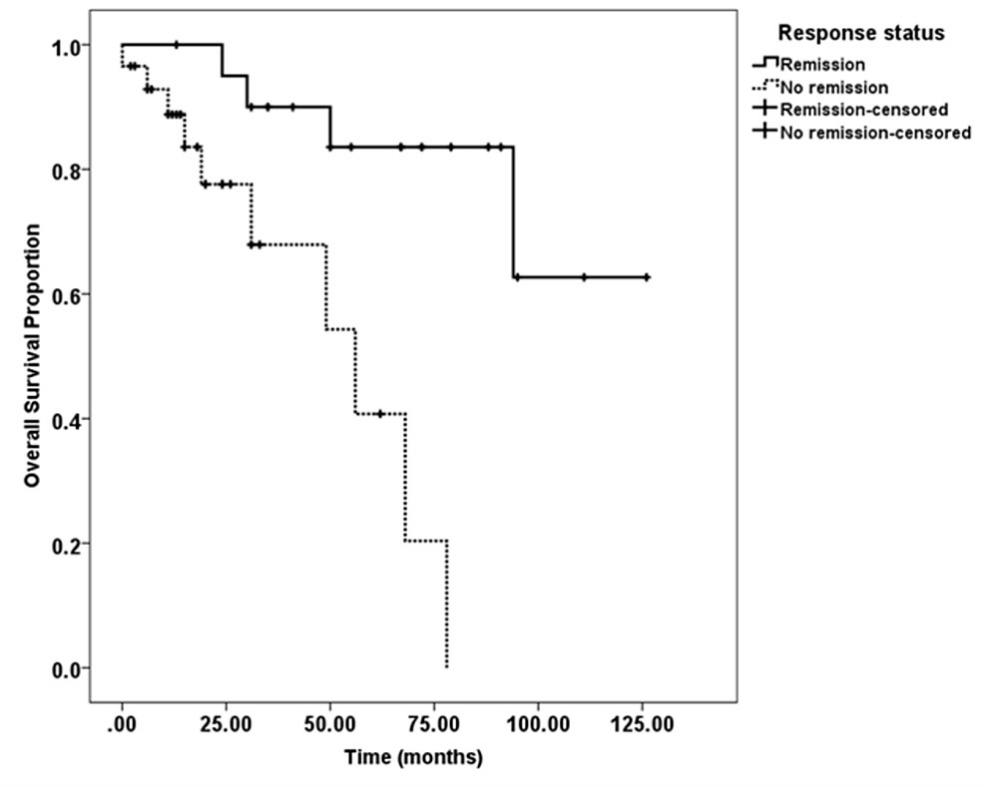
Kaplan-Meier curve of overall survival proportion of epithelial ovarian cancer patients who achieved complete radiological (radiologically no residual disease) and biochemical response (normalization of CA-125 value <21 U/ml) compared to those who were unable to get a complete response (remission).

All the patients were followed-up till death or last clinic visit, and the median survival time was 31 months, and the three-year overall survival proportion was 79%. Furthermore, there was a statistically significant difference (p-value 0.001 using log-rank test) among patients who showed complete response (remission) to initial therapy as compared to no complete response (no remission), as shown in Figure 1. Therefore, the three-year overall survival proportion in the complete response group and patients unable to achieve complete response was 90% and 68%, respectively.

## Discussion

EOC is the leading cause of cancer-related death and accounts for 3% of all cancers among women worldwide [1]. In Pakistan, ovarian cancer ranks among the ten most common cancers. EOC accounts from 63.5% to 83.3% in various studies conducted in Pakistan [11]. Increasing age, poor performance status, mucinous or clear cell histology, and gross residual disease after primary surgery are independent predictors of progression-free survival (PFS) and overall survival (OS) in advanced-stage disease as found by Winter and colleagues [8,9]. The stage at presentation is the most important predictor of survival, with five-year overall survival rates of less than 10% and a ten-year overall survival rate of only 5% [5-7]. In this study, we also looked for the predictor of long-term survival in patients of stage IV EOC who were diagnosed and treated at our centre. The present study of fifty stage IV EOC patients has been analysed. Patients who achieved complete radiological response (no residual disease on imaging) and biochemical response (normalisation of CA-125) after initial treatment showed statistically significant better long-term survival. 

Patients free of any residual disease after initial neoadjuvant chemotherapy and interval debulking surgery have strong survival benefits [14]. In our study, 29 (58%) patients received neoadjuvant platinum-based drugs and taxane chemotherapy followed by surgery, while twenty-one (42%) patients received chemotherapy but were not fit enough to undergo surgery. Among both groups, the median overall survival was thirty-one months. Literature reviews showed that the adoption of neoadjuvant chemotherapy for advanced ovarian cancer significantly reduces three years’ mortality [14,15].

Tumour histology is also a significant predictor of survival [16,17]. In our study population, the most common histological type was serous (54%), and remaining histologies were mucinous (6%), endometrioid (10%) adenocarcinoma, not otherwise specified (NOS) (10%), 2% clear cell and remaining were diagnosed on pleural and ascitic fluid analysis showing metastatic adenocarcinoma, categories as others. None of the histological subtypes was associated with statistically significant long-term survival (p-value=0.64).

The pre-treatment CA-125 value has a significant prognostic value. Higher CA-125 values are associated with an increased risk of disease progression [18]. In our study, a contrasting result was found regarding CA-125 matters. The impact of CA-125 levels behaved differently. High values of CA-125 (>1000 U/ml) at presentation were linked to better long-term survival as compared to previous studies, though it was not statistically significant (p=0.36) [19,20].

It was found that the twenty-one (42%) patients who responded and tolerated neoadjuvant platinum-based chemotherapy followed by cytoreductive surgery showed excellent outcomes. These patients achieved a complete response (normalisation of CA-125 value < 21 U/ml and radiologically no residual disease). These patients were followed up clinically every three months with CA-125 value and six-monthly CT scans. In case of recurrence after six months, platinum-based therapy was offered. If their disease relapsed earlier or remained progressive, then these patients received second-line regimens. These include gemcitabine, paclitaxel, etoposide, and topotecan [21-22]. 

Moreover, the results were also statistically significant (p-value=0.01) compared to twenty-nine patients (58%) whose disease showed either partial response, stable disease, or progressive disease. It was secondary to aggressive disease course, multiple co-morbidities, and poor ECOG performance status. Other factors associated with poor outcomes were the unavailability of drugs used in platinum-resistant disease, i.e. liposomal doxorubicin and targeted agents including bevacizumab and olaparib (PARP inhibitor) due to scarce funds [23-24].

Limitations of our study include a small number and late presentation of patients when the disease burden is very high-unavailability of drugs used in platinum-resistant disease and newer targeted agents like bevacizumab and PARP inhibitors. We also lack reporting of BRCA 1/2 mutational testing and its impact on the prediction of response to treatment and emphasise the need to identify novel approaches to management.

## Conclusions

In conclusion, advanced-stage ovarian cancer is still fatal cancer with poor outcomes. Late age at presentation with co-morbidities, impaired performance status, high-grade serous histology, and residual disease after interval debulking surgery carry worse outcomes. Achieving complete response after neoadjuvant chemotherapy, cytoreductive surgery followed by adjuvant chemotherapy has a significant prognostic impact on the patient's long-term survival. Further studies looking for patterns of gene expressions and immunological factors and their implication on patient survival and highlighting the need to identify novel approaches to managing the disease that targets the underlying biology are warranted.

## References

[1] International Agency for Research on Cancer (2019). Global cancer statistics. https://pesquisa.bvsalud.org/portal/resource/pt/lis-46560?src=similardocs.

[2] Shih IeM, Kurman RJ (2004). Ovarian tumorigenesis: a proposed model based on morphological and molecular genetic analysis. Am J Pathol.

[3] Jayson GC, Kohn EC, Kitchener HC (2014). Ovarian cancer. Lancet.

[4] Banerjee S, Kaye SB (2013). New strategies in the treatment of ovarian cancer: current clinical perspectives and future potential. Clin Cancer Res.

[5] Hamilton CA, Miller A, Casablanca Y (2018). Clinicopathologic characteristics associated with long-term survival in advanced epithelial ovarian cancer: an NRG Oncology/Gynecologic Oncology Group ancillary data study. Gynecol Oncol.

[6] McGuire WP, Hoskins WJ, Brady MF (1996). Cyclophosphamide and cisplatin compared with paclitaxel and cisplatin in patients with stage III and stage IV ovarian cancer. N Engl J Med.

[7] Ozols RF, Bundy BN, Greer BE (2003). Phase III trial of carboplatin and paclitaxel compared with cisplatin and paclitaxel in patients with optimally resected stage III ovarian cancer: a Gynecologic Oncology Group study. J Clin Oncol.

[8] Winter WE 3rd, Maxwell GL, Tian C (2007). Prognostic factors for stage III epithelial ovarian cancer: a Gynecologic Oncology Group study. J Clin Oncol.

[9] Winter WE 3rd, Maxwell GL, Tian C (2008). Tumor residual after surgical cytoreduction in prediction of clinical outcome in stage IV epithelial ovarian cancer: a Gynecologic Oncology Group study. J Clin Oncol.

[10] Bhurgi Y, Bhurgi A, Hassan SH (2000). Cancer incidence in Karachi, Pakistan: first results from Karachi Cancer Registry. Int J Cancer.

[11] Malik IA (2002). A prospective study of clinico-pathological features of epithelial ovarian cancer in Pakistan. J Pak Med Assoc.

[12] Aziz Z, Sana S, Saeed S (2003). Institution based tumor registry from Punjab: five year data based analysis. J Pak Med Assoc.

[13] Sarwar CM, Siddiqui N, Khokhar RA (2006). Epithelial ovarian cancer at a cancer hospital in a developing country. Asian Pac J Cancer Prev.

[14] Rosen B, Laframboise S, Ferguson S (2014). The impacts of neoadjuvant chemotherapy and of debulking surgery on survival from advanced ovarian cancer. Gynecol Oncol.

[15] Melamed A, Fink G, Wright AA (2018). Effect of adoption of neoadjuvant chemotherapy for advanced ovarian cancer on all cause mortality: quasi-experimental study. BMJ.

[16] Schnack TH, Høgdall E, Nedergaard L, Høgdall C (2016). Demographic clinical and prognostic factors of primary ovarian adenocarcinomas of serous and clear cell histology-a comparative study. Int J Gynecol Cancer.

[17] Ye S, Yang J, You Y (2015). Comparison of clinical characteristic and prognosis between ovarian clear cell carcinoma and serous carcinoma: a 10-year cohort study of Chinese patients. PLoS One.

[18] Zorn KK, Tian C, McGuire WP (2009). The prognostic value of pretreatment CA 125 in patients with advanced ovarian carcinoma: a Gynecologic Oncology Group study. Cancer.

[19] Kotsopoulos J, Rosen B, Fan I (2016). Ten-year survival after epithelial ovarian cancer is not associated with BRCA mutation status. Gynecol Oncol.

[20] Candido-dos-Reis FJ, Song H, Goode EL (2015). Germline mutation in BRCA1 or BRCA2 and ten-year survival for women diagnosed with epithelial ovarian cancer. Clin Cancer Res.

[21] Mutch DG, Orlando M, Goss T (2007). Randomized phase III trial of gemcitabine compared with pegylated liposomal doxorubicin in patients with platinum-resistant ovarian cancer. J Clin Oncol.

[22] Lorusso D, Sabatucci I, Maltese G, Lepori S, Tripodi E, Bogani G, Raspagliesi F (2019). Treatment of recurrent ovarian cancer with pegylated liposomal doxorubicin: a reappraisal and critical analysis. Tumori.

[23] Matulonis UA, Penson RT, Domchek SM (2016). Olaparib monotherapy in patients with advanced relapsed ovarian cancer and a germline BRCA1/2 mutation: a multistudy analysis of response rates and safety. Ann Oncol.

[24] Barber EL, Zsiros E, Lurain JR, Rademaker A, Schink JC, Neubauer NL (2013). The combination of intravenous bevacizumab and metronomic oral cyclophosphamide is an effective regimen for platinum-resistant recurrent ovarian cancer. J Gynecol Oncol.

